# Familial Clarification of Saucrosmylidae stat. nov. and New Saucrosmylids from Daohugou, China (Insecta, Neuroptera)

**DOI:** 10.1371/journal.pone.0141048

**Published:** 2015-10-20

**Authors:** Hui Fang, Dong Ren, Yongjie Wang

**Affiliations:** Key Lab of Insect Evolution and Environmental Change, College of Life Sciences, Capital Normal University, Beijing, China; Institute of Zoology, CHINA

## Abstract

**Backgound:**

Saucrosmylids are characterized by the typically large body size, complicated venation and diverse wing markings, which were only discovered in Middle Jurassic of Daohugou, Ningcheng county, Inner Mongolia, China.

**Principal Findings:**

Saucrosmylinae Ren, 2003, originally included as a subfamily in the Osmylidae, was transferred and elevated to family rank based on the definitive synapomorphic character. The updated definition of Saucrosmylidae stat. nov. was outlined in detail: presence of nygma and trichosors; diverse markings on membrane; complicated cross-veins; distal fusion of Sc and R1; expanded space between R1 and Rs having 2–7 rows of cells that should be a synapomorphic character of the family; proximal MP fork. And the previous misuses of Saucrosmylidae are also clarified. Furthermore, a new genus with a new species and an indeterminate species of Saucrosmylidae are described as *Ulrikezza aspoeckae* gen. et sp. nov. and *Ulrikezza* sp. from the Middle Jurassic of Daohugou, Inner Mongolia, China. A key to genera of Saucrosmylidae is provided.

**Conclusions/Significance:**

The intriguing group represents a particular lineage of Neuroptera in the Mesozoic Era. The familial status of Saucrosmylidae was firstly advanced that clarified the former incorrect citation and use of the family name. As an extinct clade, many species of the saucrosmylids were erected just based on a single fore- or hindwing, and it should be realized that providing more stable characters is necessary when describing new lacewing taxa just based on an isolated hindwing. It is vital for the systematics of Saucrosmylidae.

## Introduction

The Saucrosmylidae stat. nov., an extraordinary group of Neuroptera, is only discovered in Mesozoic currently. Up to now, six genera from the Middle Jurassic of Daohugou, Inner Mongolia, China, have been classified into Saucrosmylidae, including *Saucrosmylus* Ren & Yin, 2003 [1]; *Rudiosmylus* Ren & Yin, 2003 [1]; *Laccosmylus* Ren & Yin, 2003 [1]; *Bellinympha* Wang et al., 2010 [2]; *Huiyingosmylus* Liu et al., 2013 [3] and *Daohugosmylus* Liu et al., 2014 [4]. Although only six genera were formally published, saucrosmylids show the significant high morphological diversity, and the oldest pinnate insect was also recorded from the family that implying its unusual environmental adaptation in the Mesozoic [2].

The enigmatic insect was firstly established as a subfamily of Osmylidae for sharing the most plesiomorphies, e.g. presence of nygma; presence of trichosors; Sc and R1 fused apically and entering margin before wing apex [1]. However, it possesses the distinctive apomorphic characters that differ from Osmylidae, e.g. notably dense venation and widely space between R1 and Rs producing 2–7 rows of cells that should be a synapomorphic character of Saucrosmylidae. While the complicated venation within Saucrosmylidae suggested that the particular insects likely have the close relationships to its Mesozoic affinities Kalligrammatidae, Grammolingiidae, Aetheogrammatidae, Panfiloviidae and Parakseneuridae instead of Osmylidae [5–11]. However, the exceptionally broad space between R1 and Rs can easily separate Saucrosmylidae from their Mesozoic affinities. Therefore it is suitable to elevate Saucrosmylidae to an independent family branched off from Osmylidae. And a detailed diagnosis of the family was provided: antennae filiform, forewing slender, diverse wing markings, dense venation, costal cross-veins complex and usually interlinked by numerous veinlets, space between R1 and Rs expand at middle part with many cross-veins producing several rows of cells, Rs curved distally, MA originated from Rs and distal forked, MP forked close to wing base, CuA long and pectinately branched, CuP short and usually forming deep dichotomous forks, 1A and 2A well-developed. We also presented a key to genera of the family.

Furthermore, a new genus with a new species and an indeterminate species of Saucrosmylidae are described as *Ulrikezza aspoeckae* gen. et sp. nov. and *Ulrikezza* sp. from the Jiulongshan Formation at Daohugou Village, Shantou Township, Ningcheng County, Inner Mongolia, China. The Daohugou strata are famous for the richness of a diverse fauna especially for the complete and excellent insect specimens [9, 12–16]. The Daohugou stratigraphic sequence is controversial for a long time [17–22]. Based on the current stage of knowledge, the Daohugou strata should be considered Middle to Late Middle Jurassic in age [9, 14, 23].

## Material and Methods

All photographs were taken using a Nikon D90 digital camera. The line drawing was prepared on photographs using the image-editing software CorelDRAW X6. Terminology follows New (1983) [24]. Wing vein abbreviations are as follows: C, costa; Sc, subcosta; R, radius; R1, first branch of R; Rs, radial sector; M, media; MA, media anterior; MP, media posterior; Cu, cubitus; CuA, cubitus anterior; CuP, cubitus posterior; 1A–3A, first to third anal veins. The specimens described in this paper are deposited in the Key Lab of Insect Evolution & Environment Change, College of Life Sciences, Capital Normal University.

## Results

### Systematic palaeontology

Class Insecta Linnaeus, 1758

Order Neuroptera Linnaeus, 1758

### Family Saucrosmylidae stat. nov

#### Type genus

*Saucrosmylus* Ren & Yin, 2003

#### Included genera

*Saucrosmylus* Ren & Yin, 2003; *Rudiosmylus* Ren & Yin, 2003; *Laccosmylus* Ren & Yin, 2003; *Bellinympha* Wang et al., 2010; *Huiyingosmylus* Liu et al., 2013; *Daohugosmylus* Liu et al., 2014; *Ulrikezza* gen. nov.

#### Diagnosis

Large insects (wing exceeding 60mm in length generally); antennae filiform, much shorter than the half of forewing in length; forewing slender, and forming undulated margin in some particular groups; presence of nygma; presence of trichosors; membrane commonly with varied fuscous markings; venation extraordinarily complicated and dense comparing to the extant neuropterans; costal cross-veins forming various distal forks or complex secondary forks, and usually interlinked by numerous veinlets; Sc and R1 fused apically and thence curved posteriad to enter margin before wing apex; area between R1 and Rs widely spaced, and producing 2–7 rows of cells; Rs with several main branches before Rs curved and bent anteriorly distally, each with complicated distal forks and angled toward posterior apical margin; MA originated from Rs, possessing complicated distal branches; MP fork close to wing base, usually between separations of MA and the first branch of Rs; CuA and CuP commonly diverged at the wing base; CuA pectinately branched from the midway; CuP generally forming the deep dichotomous forks; anal region broad, A1 parallel to the posterior margin, with many primary branches; hindwing generally slender, resembling the forewing, but in some genera the shape of hindwing heterogeneous to the forewing.

#### Comments

Saucrosmylidae stat. nov. is an extinct and intriguing family of the Mesozoic Neuroptera, characteristic of the remarkably large body size, dense venation and the impressive wing markings. It was firstly described as a subfamily Saucrosmylinae of Osmylidae by Ren and Yin in 2003 [1], and then six genera were erected including the oldest pinnate leaf mimesis insects *Bellinympha* [2]. Actually the original assignment to Osmylidae was just based on the symplesiomorphies of Neuroptera: presence of nygma; antennae filiform, much shorter than half forewing length; presence of trichosors; Sc and R1 fused apically and entering margin before wing apex; membrane with microtrichia; between R1 and Rs area with numerous cross-veins; origin of Rs close to the base of wing [1].

In 2010, Wang asserted saucrosmylids possessed the apparent apomorphic characters which were easily separated from other families, and it was proper to elevate it to family rank in his dissertation [25]. This work had never been formally published before, and so Saucrosmylidae should be an unavailable family name according to the ICZN. However, some researchers cited the abovementioned work and incorrectly regarded it as a formal family in their studies. In 2012, Yang et al. treated Saucrosmylidae as an independent family in their phylogenetic analysis on Neuroptera [9]. Although this use of the family was not proper, ‘Saucrosmylidae’ clearly did not group with Osmylidae and entered psychopsid-clade with five extinct families: Kalligrammatidae, Grammolingiidae, Aetheogrammatidae, Panfiloviidae and Parakseneuridae. In despite of the uncertain interrelationships within Psychopsoid clade, it evidently implied Saucrosmylidae should be an independent group that was distant from Osmylidae. In 2013 Liu et al. established a new genus *Huiyingosmylus* from China, and they correctly treated Saucrosmylinae as a subfamily of Osmylidae [3]. Subsequently, while they described another genus *Daohugosmylus*, they falsely began to use Saucrosmylidae instead of Saucrosmylinae [4]. In fact, the familial status of Saurosmylidae should be invalid before it is formally elevated to family rank, therefore it is necessary to re-clarify the vague position of this group.

In comparison to other contemporaneous neuropteran families, saurosmylids shared the distinctive synapomorphies that corroborate its familial status: remarkably large body size; notably dense venation that suggests the close relationships to its Mesozoic affinities Kalligrammatidae, Grammolingiidae, Aetheogrammatidae, Panfiloviidae and Parakseneuridae [5–11]; widely spaced R1 and Rs area, producing 2–7 rows of cells; CuA forming a large triangular region. Kalligrammatidae are known as “butterflies of the Jurassic” [26], with forewing exceptionally broader than Saucrosmylidae, and they can be easily recognized by the apomorphic character that MP extensively branched, MP2 with many distal pectinate branches, forming a large triangular region (except for *Sophogramma*) [5]. Grammolingiidae shows some superficial appearance similarity with Saucrosmylidae (especially in *Saucrosmylus*), however dissociation of Sc and R1 in Grammolingiidae implies the both families should represent the independent evolutionary lineages. Aetheogrammatidae was only found in the Early Cretaceous strata, and it could be easily separated from Saucrosmylidae by the shape of wing, and absences of trichosors and microtrichia. Panfiloviidae have extremely more complicated venation than Saucrosmylidae; space between Sc and R1 possesses numerous cross-veins, while in Saucrosmylidae, only one strip of cross-veins presents between Sc and R1 [8]. Costal area of Parakseneuridae is from basally broad to distally narrow, while the space is uniformity from base to ends in Saucrosmylidae; costal cross-vein is more complicated; CuA forms relatively simple dichotomous branches, not forming a large triangular area, which is very different from Saucrosmylidae [9]. Considering the actual differences of Saucrosmylidae with other families, it is suitable to elevate it to a family rank.

### Key to the genera of Saucrosmylidae

1. Wing with outer margin strongly undulate…………………………………………2
  Wing with outer margin slightly undulate …………………………………………3
2. Spotted markings on the wing surface; Rs slightly bent anteriorly towards R1…….…………………………………….………………………*Huiyingosmylus*
  Pinna-like markings on the wing surface; Rs sharply bent anteriorly towards R1…….…………………………………….…………………………*Bellinympha*
3. Rs sharply bent anteriorly towards R1…….…………………………………….…4
  Rs slightly bent anteriorly towards R1…….…………………………………….…5
4. Wing with at most 6–7 rows of irregular cells in R1 area……….………*Laccosmylus*
  Wing with 3 rows of regular cells in R1 area ……….……………… *Saucrosmylus*
5. Costal cross-veins in hindwing nearly not forked; CuP simply formed 2–3 dichotomous branches………………….…………………………*Ulrikezza* gen. nov.
  Costal cross-veins in hindwing forked; CuP formed 5–8 pectinate branches……………………………………………………………………………6
6. Stripy markings and several fuscous and subcircular spots on the wing surface.……………………………………………………………… *Daohugosmylus*
  Spotted markings on the wing surface…………………….…………… *Rudiosmylus*

### Genus *Ulrikezza* gen. nov

#### Type species

*Ulrikezza aspoeckae* gen. et sp. nov.

#### Etymology

The generic name is dedicated to the outstanding neuropterist Prof. Ulrike Aspöck for her tremendous contributions to the research of Neuroptera.

#### Diagnosis

Large insect, forewing slender and membrane decorated with 7 fuscous eye spots and some small spotted markings; outer margin smooth, decorated with trichosors and microtrichia; costal cross-veins relatively simple, with shallow distal forks; space between R1 and Rs with 4–5 rows of irregular cells; MA originated from Rs close to wing base, MP forked between separation of MA and Rs1 from Rs; CuA and CuP deeply forked, CuA forked at midwing, forming complicated pectinate branches, CuP short and relatively simple, formed 2–3 distal dichotomous branches. Hindwing distinctively broader comparing to the forewing; costal cross-veins single branch, or occasionally with distal forks.

#### Comment

*Ulrikezza* gen. nov. showing typical saucrosmylid-like appearances is unequivocal attributed to Saucrosmylidae. Because *Ulrikezza* gen. nov. is founded based on the complete specimen preserved with four wings, it could conduct the comprehensive comparisons to the other genera. It can be distinguished from the type genus *Saucrosmylus* by the following hindwing characters: the wings have spotted markings vs stripy markings in *Saucrosmylus*; R1 space has 4–5 rows of irregular cells vs three regular rows of cells; Rs slightly bent anteriorly towards R1 vs Rs sharply bent anteriorly towards R1. It can be easily distinguished from forewing of *Bellinympha* through its spotted marking, slightly bent Rs and smooth outer margin, while *Bellinympha* has pinna-like markings on the wing surface, sharply bent Rs and outer margin strongly undulate. The new genus differs from *Huiyingosmylus* by the following forewing characters: much slender wing shape and smooth outer margin in the former, but broader wing and outer margin strongly undulate in the latter; MA and Rs1 fused at outer margin distally in the former, but Rs1 and Rs2 fused at two-third of the wing in the latter; the curved CuA in the latter form a more large area than in the former.

For other genera erected by the hindwing, *Ulrikezza* still could be detected some significant differences to divide them. *Ulrikezza* shares some most similarities with *Rudiosmylus* that was established by Ren in 2003, however they could be easily distinguished by the figuration of costal cross-veins: costal cross-veins single branch or occasional distal forked vs most costal cross-veins with distal forks; costal cross-veins straight or slightly sinuous in the new genus vs costal cross-veins conspicuously sinuate at base in *Rudiosmylus*; numerous veinlets between costal cross-veins in the new genus (5–7 rows), but only a few veinlets between costal cross-veins in the latter (2–4 rows); CuP formed 2–3 pectinate branches in the former, while CuP formed 8 pectinate branches in the latter. *Ulrikezza* gen. nov. differs from hindwing of *Laccosmylus* in having a much slender hindwing with smooth outer margin (slightly undulate in *Laccosmylus*). *Ulrikezza* gen. nov. can be easily distinguished from *Daohugosmylus* by the following hindwing characters: the wings have spotted markings in the former, but stripes and spots in the latter; the R1 space has 4–5 rows of irregular cells in the former, but only three regular rows in the latter; numerous cross-veins between costal veinlets in the former, but only a few veinlets between costal cross-veins in the latter; one row of cells between MA and Rs1 in the former, but two rows of cells in the latter; one row of cells between CuA and MP2 in the former, but two rows of cells in the latter; the former has less CuA branches than the latter.

### *Ulrikezza aspoeckae* sp. nov

#### Etymology

Refer to the generic name.

#### Diagnosis

In forewing, MA coalescent with Rs1 or connect through a thick vein distally; CuA with 6 or 9 branches; space between the furcation of CuP only possess one cell; in hindwing, costal veinlets nearly not forked or only individual forked and 5–7 rows of veinlets between costal cross-veins.

#### Holotype

**Fig 1:**
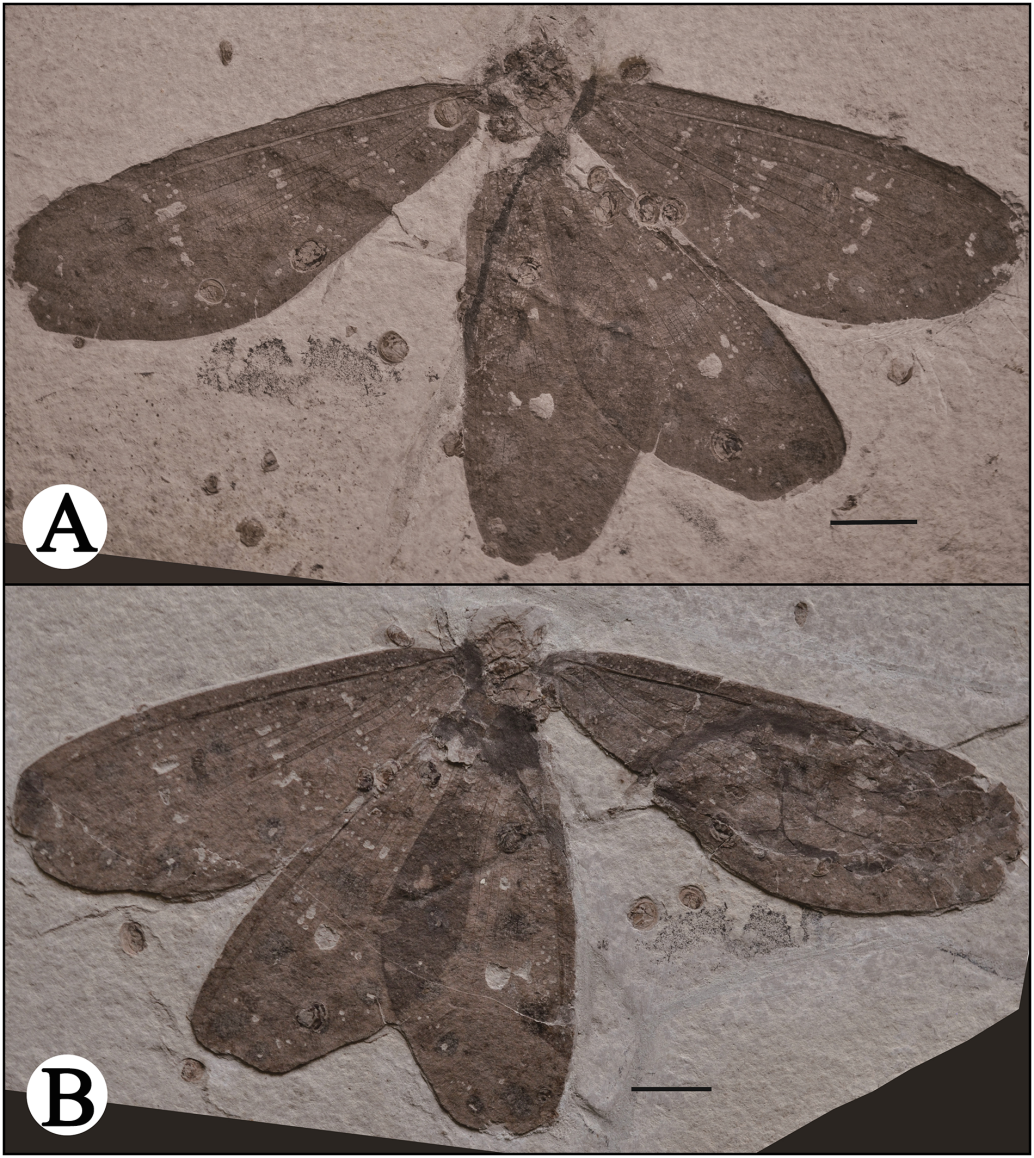
*Ulrikezza aspoeckae* gen. et sp. nov Holotype: CNU-NEU-NN2015001PC. A. part, B. counterpart; Scale bar = 10 mm.

CNU-NEU-NN2015001PC, well-preserved wings with apical part of each wing lacking, and a seriously deformed protothorax (Fig 1).

#### Description

**Fig 2:**
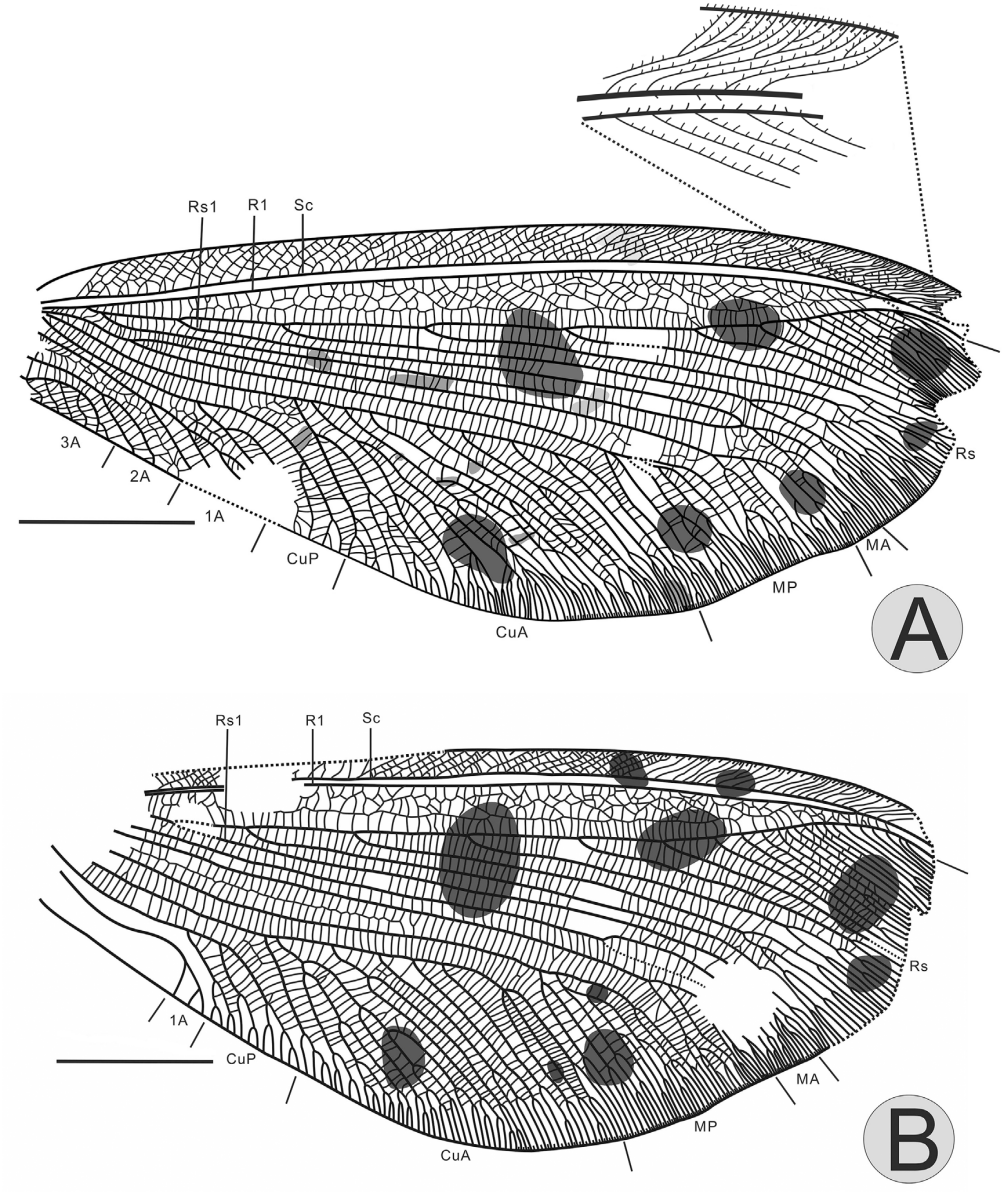
*Ulrikezza aspoeckae* gen. et sp. nov Line drawings of complete venation for right forewing (A) and right hindwing (B). Scale bar = 10 mm.

**Fig 3:**
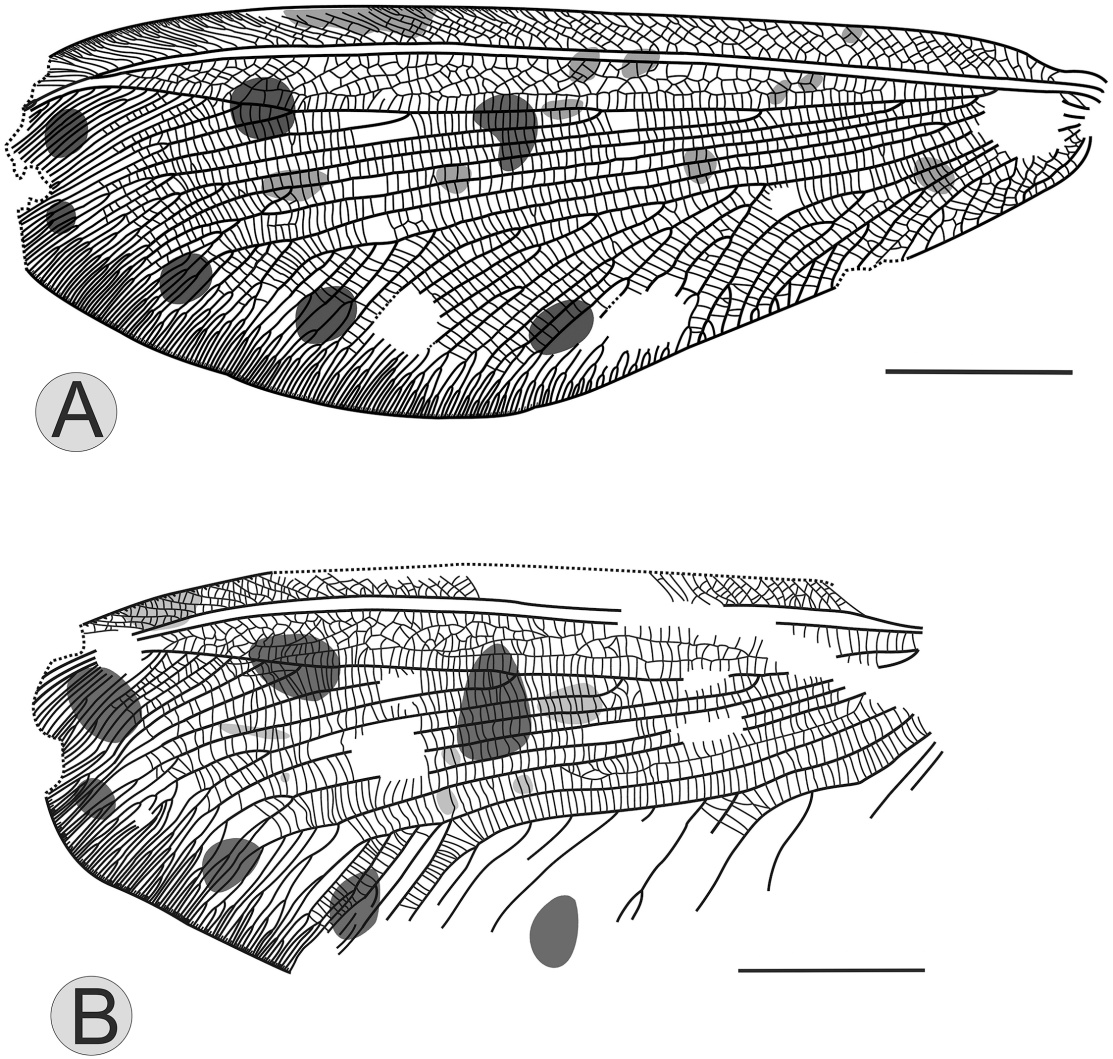
*Ulrikezza aspoeckae* gen. et sp. nov Line drawing of complete venation for left forewing (A) and left hindwing (B). Scale bar = 10 mm.

Forewing about 57mm long and 22mm wide, apex missing (Figs 2A and 3A). Trichosors and nygma present. Outer margin relatively smooth. Costal space basally very narrow, then strongly expanded. Costal cross-veins forked distally, interlinked by numerous veinlets. Sc fused with Rl apically and ending on costal margin before wing apex. Cross-veins sc-r1 absent. Rs slightly bent anteriorly towards R1; space between Rs and R1 broad, forming 4–5 rows of irregular cells; Rs with six main branches before Rs bent anteriorly, each with complicated distal forks and angled toward posterior apical margin. In right forewing, MA coalescent with Rs1; while MA is connected with Rs1 through a thick vein in left forewing. MP forked between separations of MA and Rs1 from Rs. CuA with numerous oblique pectinate branches forked distally at about one fourth length of wing, forming large triangular area. CuA forked 9 branches in right forewing while 6 branches in left forewing, the performance of the first branch of CuA is bold in each wing. CuP short and simple with only two main branches, forked slightly basad of CuA. Three anal veins well preserved. 1A with numerous regular and oblique pectinate branches forked at about one fourth length of the vein. Two rows of cells between 1A and 2A. In right forewing, 2A forms simple dichotomy; while in left forewing, 2A with numerous pectinate branches forked basally; 3A short.

Hindwing somewhat shorter than forewing, about 48mm long and 21mm wide, apex missing and overlapping partially (Figs 2B and 3B). Venation is similar to the forewing. More than 6 rows of cross-veins between costal veinlets which are rather dense but only individual forked. MA barely forked tree branches distally. MP forked close to wing base. A couple rows of cells between MP1 and MP2. CuA with 7 oblique pectinate branches forked distally at about one fourth length of wing, forming large triangular area. CuP short, with 3 oblique pectinate branches, forked slightly basad of CuA. Anal region is small, 1A curved.

#### Type location and horizon

Daohugou Village, Wuhua Township, Ningcheng County, Chifeng City, Inner Mongolia, China; Jiulongshan Formation, Middle Jurassic.

### *Ulrikezza* sp

#### Material Examined

**Fig 4:**
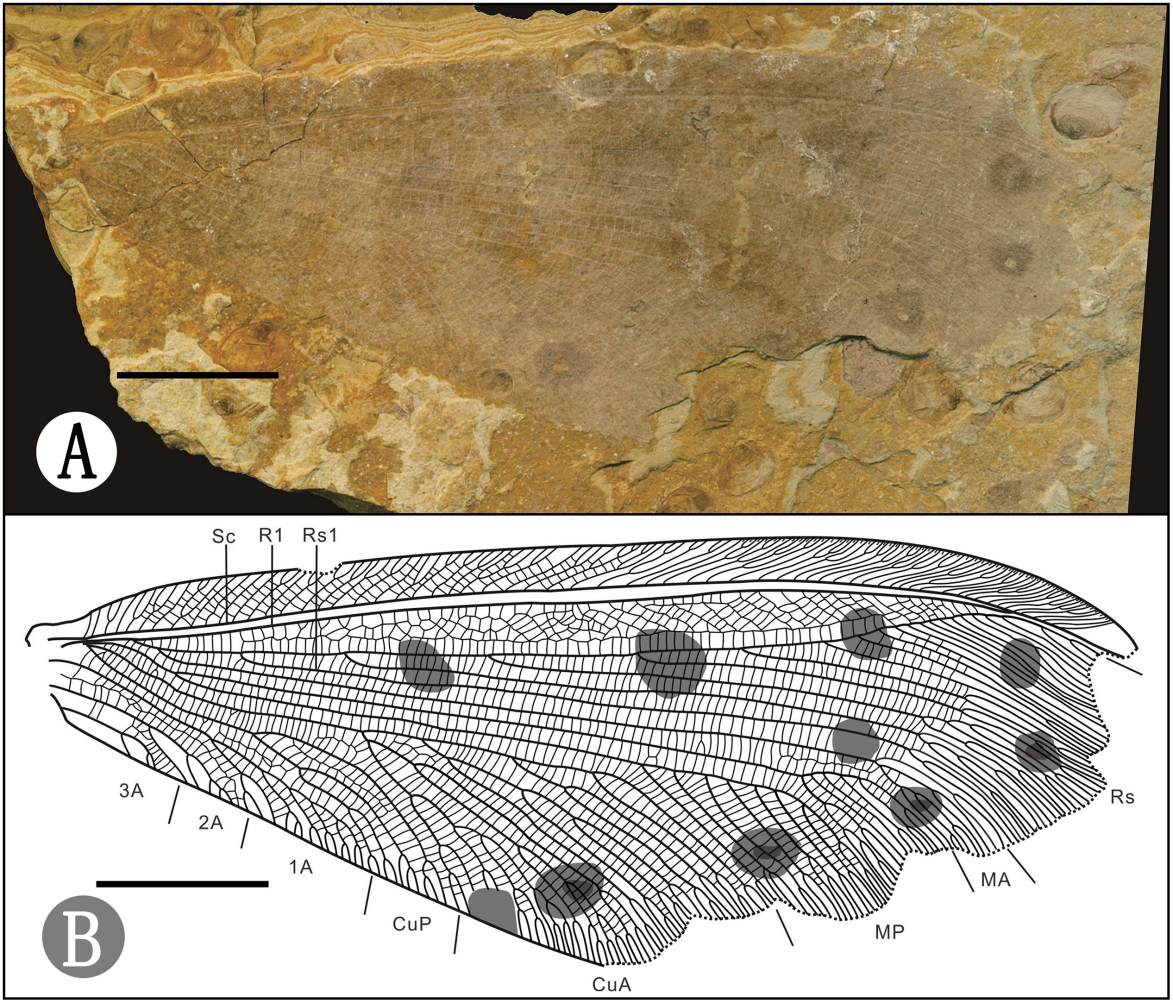
*Ulrikezza* sp., CNU-NEU-NN2015002 A. Photograph; B. Outline of holotype for the forewing. Scale bar = 10 mm.

CNU-NEU-NN2015002, only a single forewing is preserved, with apex missing and outer margin partially lacking (Fig 4A).

#### Description

Forewing 63.7mm long and 25.7mm wide, outer margin missing. Dark spotted markings distribute on the slender forewing (Fig 4B). Costal space is very narrow basally, then strongly expanded. Costal cross-veins forked distally, numerous veinlets merely distribute basally and medially. Sc fused with Rl apically and ending on costal margin before wing apex. Rs slightly bent anteriorly towards R1; space between Rs and R1 forming 6–7 rows of irregular cells, arranged obliquely on the edge of the space. MA as a single branch is forked distally. MP forked between separations of MA and Rs1 from Rs, MP1 formed tree branches, MP2 formed pectinate branches. CuA with four complex pectinate branches forked distally at about one fourth length of wing, forming large triangular area. CuP short and simple with only two main branches forming two rows of cells. Three anal veins well preserved. 1A with numerous regular and oblique pectinate branches forked at about one fourth length of the vein. Two rows of cells between 1A and 2A. 3A short.

#### Type location and horizon

Same as those of *U*. *aspoeckae*.

#### Comment

The venation of this specimen is quite similar to that of *U*. *aspoeckae*; however, they have some relatively obvious differences: MA in this specimen separate from Rs1, while in *U*. *aspoeckae*, MA coalescent with Rs1 or connect through a thick vein distally; CuA forked five branches in this specimen, while 6 or 9 branches in *U*. *aspoeckae*; space between the first and the second branches of CuA possess two rows of cells, while in *U*. *aspoeckae* there is just one row of cells; two rows of cells between the two branches of CuP, while just one row of cells between the furcation of CuP in *U*. *aspoeckae*. In spite of the differences between the specimen and *U*. *aspoeckae*, we consider it is not suitable to describe a new species only based on a single wing. Therefore we treat it as an uncertain species until the further specimen could be found.

## Discussion

Up to date, Saucrosmylidae have been only found from the Jiulongshan Formation of China [1–4]. The eye-catching large insects showed the particularly morphological diversity and specific richness. Due to the limiting condition of preservation, a common thing to the researcher is a new taxon was usually built based on the partial specimens that possibly resulted in the incomplete identified data of the new taxon. And some taxon was established by the single wing (e.g. *Rudiosmylus* Ren & Yin, 2003 [1]; *Laccosmylus* Ren & Yin, 2003 [1]; *Huiyingosmylus* Liu et al., 2013 [3] and *Daohugosmylus* Liu et al., 2014 [4]). The condition is normal among the fossil insects especially the large insects, Kalligrammatidae and Grammolingiidae. Although fore- and hindwing show some similarities in wing shape and venation, some students overlooked the factual discrepancies of the both wings. Even some researchers likely mistook the fore- and hindwing and resulted in a false decision. In 2014, Yang et al pointed that wings of *K*. *jurarchegonium* and *K*. *liaoningense* described in original literature actually should be hindwings [5]. These mistakes baffled the subsequent taxonomic and phylogenetic work, because the differences came from the heterogenous fore- and hindwing instead of the anticipated same wing. Occurrence of *Ulrikezza* that possessed the different shape of fore- and hindwings implies saucrosmylids also have the potential confusion of the both wings similar to Kalligrammatidae. Herein we outlined the features of fore- and hindwing in Saucrosmylidae in case of the potential misunderstandings of them. The both wings can be distinguished by the following characters: forewing vein MP forked near base of wing, usually between separations of MA and Rs1 from Rs, while hindwing vein MP forked basally and usually before separations of MA and Rs; forewing is often a little longer than hindwing; sometimes, costal veinlets with few dichotomy in the hindwing, but a number of dichotomies in the forewing; in some genera, forewing spots are more obvious than hindwing or different from hindwing, e.g. in *Bellinympha*, pinna-like markings only occurred on the forewing; one row of cells between MP1 and MP2 in the forewing, but often a couple rows of cells between MP1 and MP2 in the hindwing; A1 in forewing generally parallel to the posterior margin, while in hindwing A1 is usually bent; in addition, forewing is a little different from hindwing in shape, the number of Rs branches and the number of CuA and CuP branches.

Ideally the complete description is preferred when a new taxon was established, while it usually became an impossible task in studying the fragmentary insect fossil. It would be much more important to correctly distinguish the both wings when the distinctive discrepancies occurring at both fore- and hindwing. Similar to *Ulrikezza*, hindwing sometimes possesses the same shape and same markings, even a close relative of veination to forewing, which leads misjudgement much more likely to happen. Therefore, pointing out the differences between fore- and hindwing as the feasible method can play an important role in the follow-up study of Saucrosmylidae. It is important to emphasize that, as the research progressed, some new genera and new species have a serious possibility in possessing huge disparity between fore- and hindwing, like in *Bellinympha*. If this happens, building a new taxon only based on a single hindwing is not advisable; especially when its forewing is found, it is difficult to relate with the hindwing, this situation may lead to the faulty practice of establishing another new taxon. According to the existence of differences between fore- and hindwing, it should be more cautious to describe a new taxon when only a single hindwing was found. Also, a comprehensive comparison is necessary to reduce the synonyms.

## Conclusions

This paper clarifies the previous misuses of Saucrosmylidae, formally elevates Saucrosmylinae to family rank, gives full descriptions about a new genus with a new species and an indeterminate species of Saucrosmylidae (*Ulrikezza aspoeckae* gen. et sp. nov. and *Ulrikezza* sp.) from the Middle Jurassic of Daohugou, Inner Mongolia, China, and provides a key to genera of Saucrosmylidae. In addition, the discrepancies between fore- and hindwing within Saucrosmylidae were preliminarily summarized, and it was pointed out that it should provide more stable characters in case of mistakes and misleading when describing new lacewing taxa just based on an isolated hindwing.
